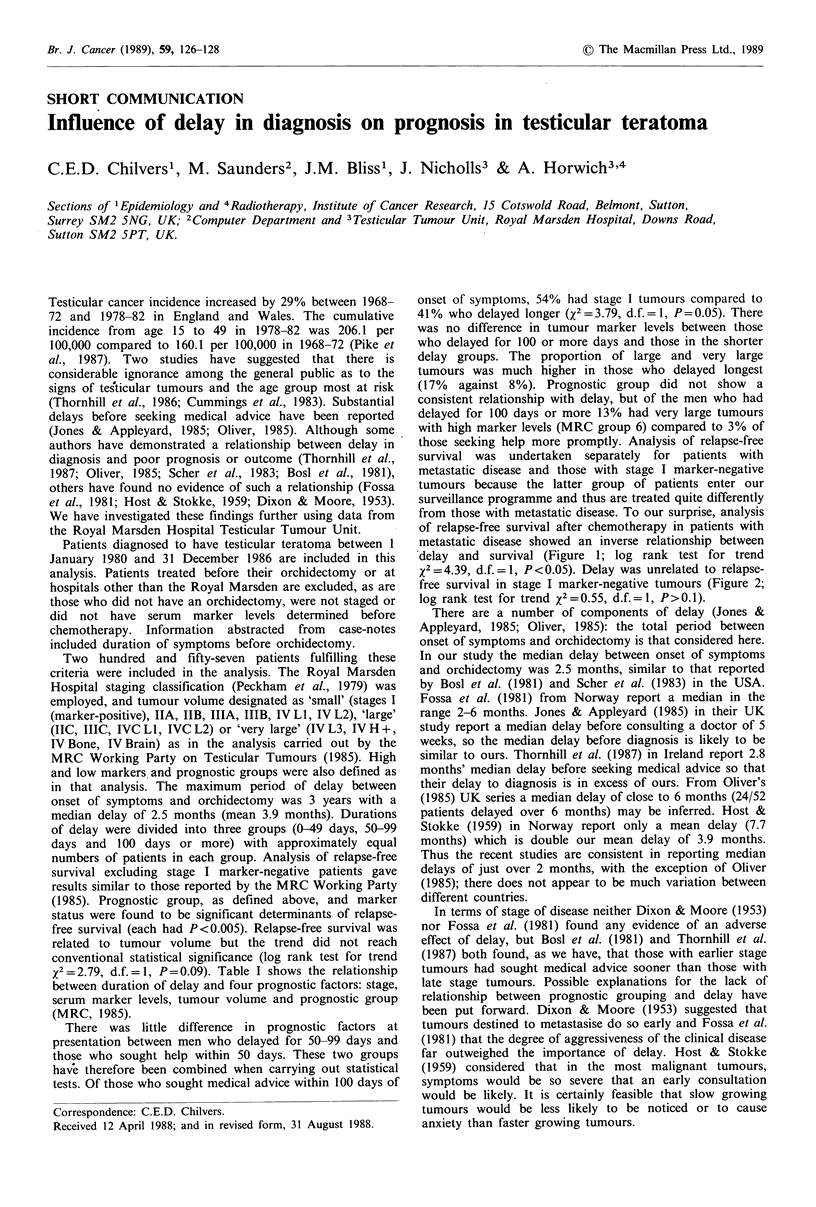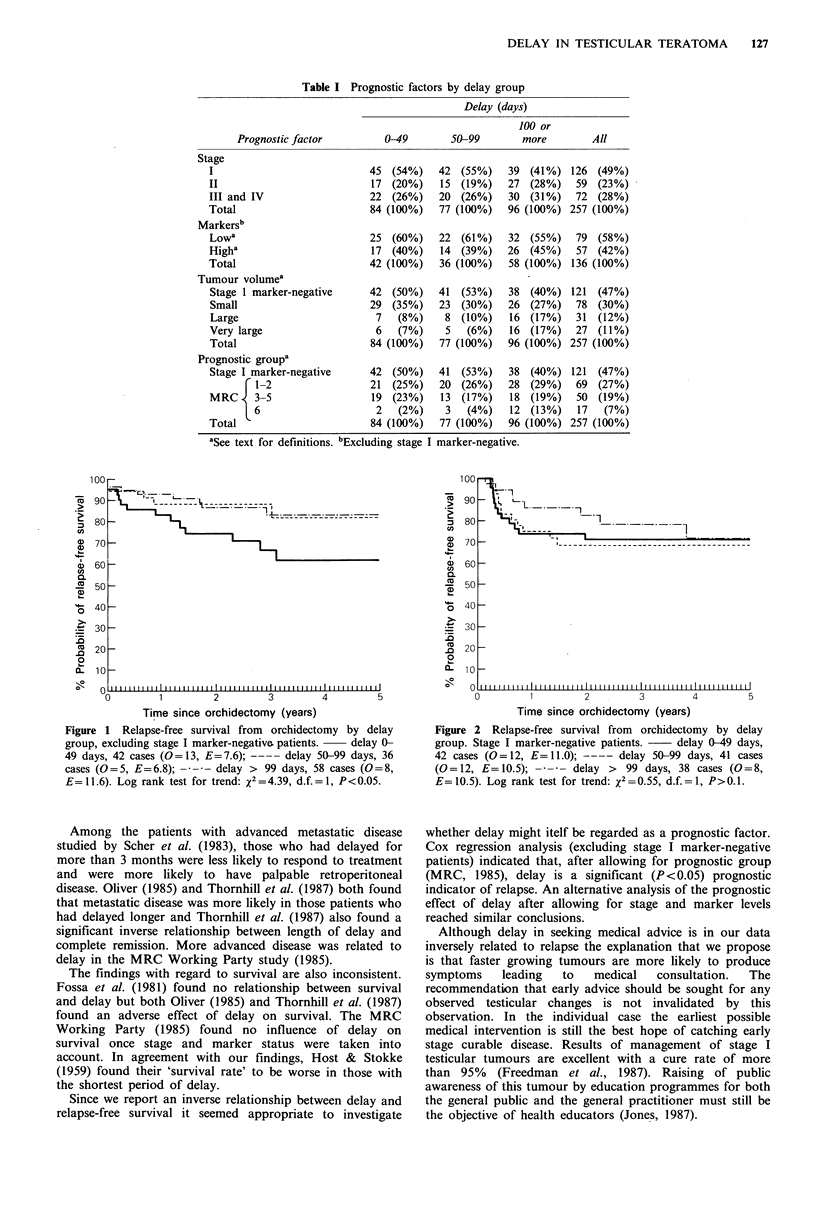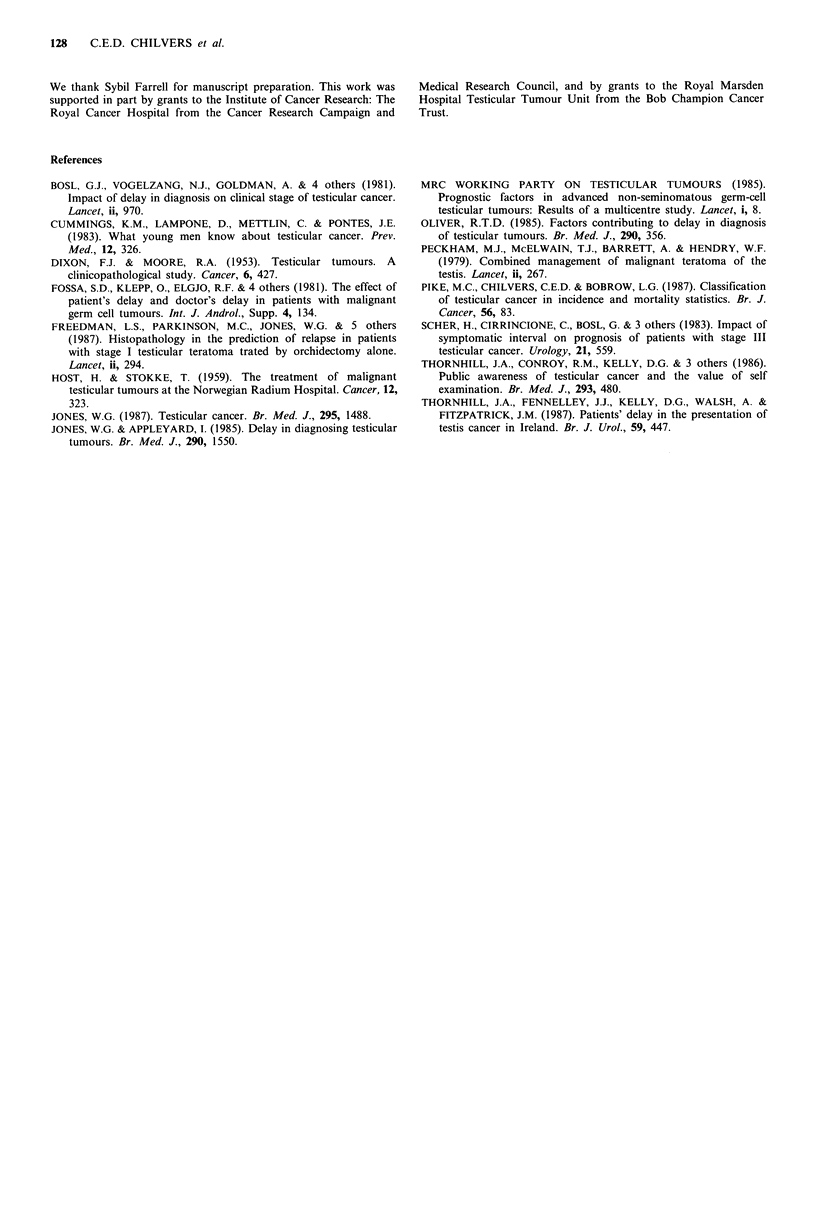# Influence of delay in diagnosis on prognosis in testicular teratoma.

**DOI:** 10.1038/bjc.1989.25

**Published:** 1989-01

**Authors:** C. E. Chilvers, M. Saunders, J. M. Bliss, J. Nicholls, A. Horwich

**Affiliations:** Section of Epidemiology, Institute of Cancer Research, Sutton, Surrey, UK.


*Br. J. Cancer* (1989), **59**, 126–128 © The Macmillan Press Ltd., 1989

SHORT COMMUNICATION

### Influence of delay in diagnosis on prognosis in testicular teratoma

C.E.D. Chilvers[1], M. Saunders[2], J.M. Bliss[1], J. Nicholls[3] & A. Horwich[3,4]

*Sections of [1]Epidemiology and [4]Radiotherapy, Institute of Cancer Research, 15 Cotswold Road, Belmont, Sutton, Surrey SM2 5NG, UK; [2]Computer Department and [3]Testicular Tumour Unit, Royal Marsden Hospital, Downs Road, Sutton SM2 5PT, UK.*

Testicular cancer incidence increased by 29% between 1968–72 and 1978–82 in England and Wales. The cumulative incidence from age 15 to 49 in 1978–82 was 206.1 per 100,000 compared to 160.1 per 100,000 in 1968–72 (Pike *et al.*, 1987). Two studies have suggested that there is considerable ignorance among the general public as to the signs of testicular tumours and the age group most at risk (Thornhill *et al.*, 1986; Cummings *et al.*, 1983). Substantial delays before seeking medical advice have been reported (Jones & Appleyard, 1985; Oliver, 1985). Although some authors have demonstrated a relationship between delay in diagnosis and poor prognosis or outcome (Thornhill *et al.*, 1987; Oliver, 1985; Scher *et al.*, 1983; Bosl *et al.*, 1981), others have found no evidence of such a relationship (Fossa *et al.*, 1981; Host & Stokke, 1959; Dixon & Moore, 1953). We have investigated these findings further using data from the Royal Marsden Hospital Testicular Tumour Unit.

Patients diagnosed to have testicular teratoma between 1 January 1980 and 31 December 1986 are included in this analysis. Patients treated before their orchidectomy or at hospitals other than the Royal Marsden are excluded, as are those who did not have an orchidectomy, were not staged or did not have serum marker levels determined before chemotherapy. Information abstracted from case-notes included duration of symptoms before orchidectomy.

Two hundred and fifty-seven patients fulfilling these criteria were included in the analysis. The Royal Marsden Hospital staging classification (Peckham *et al.*, 1979) was employed, and tumour volume designated as 'small' (stages I (marker-positive), IIA, IIB, IIIA, IIIB, IV L1, IV L2), 'large' (IIC, IIIC, IVC L1, IVC L2) or 'very large' (IV L3, IV H+, IV Bone, IV Brain) as in the analysis carried out by the MRC Working Party on Testicular Tumours (1985). High and low markers and prognostic groups were also defined as in that analysis. The maximum period of delay between onset of symptoms and orchidectomy was 3 years with a median delay of 2.5 months (mean 3.9 months). Durations of delay were divided into three groups (0–49 days, 50–99 days and 100 days or more) with approximately equal numbers of patients in each group. Analysis of relapse-free survival excluding stage I marker-negative patients gave results similar to those reported by the MRC Working Party (1985). Prognostic group, as defined above, and marker status were found to be significant determinants of relapse-free survival (each had $P<0.005$). Relapse-free survival was related to tumour volume but the trend did not reach conventional statistical significance (log rank test for trend $\chi^2=2.79$, d.f.$=1$, $P=0.09$). Table I shows the relationship between duration of delay and four prognostic factors: stage, serum marker levels, tumour volume and prognostic group (MRC, 1985).

There was little difference in prognostic factors at presentation between men who delayed for 50–99 days and those who sought help within 50 days. These two groups have therefore been combined when carrying out statistical tests. Of those who sought medical advice within 100 days of onset of symptoms, 54% had stage I tumours compared to 41% who delayed longer ($\chi^2=3.79$, d.f.$=1$, $P=0.05$). There was no difference in tumour marker levels between those who delayed for 100 or more days and those in the shorter delay groups. The proportion of large and very large tumours was much higher in those who delayed longest (17% against 8%). Prognostic group did not show a consistent relationship with delay, but of the men who had delayed for 100 days or more 13% had very large tumours with high marker levels (MRC group 6) compared to 3% of those seeking help more promptly. Analysis of relapse-free survival was undertaken separately for patients with metastatic disease and those with stage I marker-negative tumours because the latter group of patients enter our surveillance programme and thus are treated quite differently from those with metastatic disease. To our surprise, analysis of relapse-free survival after chemotherapy in patients with metastatic disease showed an inverse relationship between delay and survival (Figure 1; log rank test for trend $\chi^2=4.39$, d.f.$=1$, $P<0.05$). Delay was unrelated to relapse-free survival in stage I marker-negative tumours (Figure 2; log rank test for trend $\chi^2=0.55$, d.f.$=1$, $P>0.1$).

There are a number of components of delay (Jones & Appleyard, 1985; Oliver, 1985): the total period between onset of symptoms and orchidectomy is that considered here. In our study the median delay between onset of symptoms and orchidectomy was 2.5 months, similar to that reported by Bosl *et al.* (1981) and Scher *et al.* (1983) in the USA. Fossa *et al.* (1981) from Norway report a median in the range 2–6 months. Jones & Appleyard (1985) in their UK study report a median delay before consulting a doctor of 5 weeks, so the median delay before diagnosis is likely to be similar to ours. Thornhill *et al.* (1987) in Ireland report 2.8 months' median delay before seeking medical advice so that their delay to diagnosis is in excess of ours. From Oliver's (1985) UK series a median delay of close to 6 months (24/52 patients delayed over 6 months) may be inferred. Host & Stokke (1959) in Norway report only a mean delay (7.7 months) which is double our mean delay of 3.9 months. Thus the recent studies are consistent in reporting median delays of just over 2 months, with the exception of Oliver (1985); there does not appear to be much variation between different countries.

In terms of stage of disease neither Dixon & Moore (1953) nor Fossa *et al.* (1981) found any evidence of an adverse effect of delay, but Bosl *et al.* (1981) and Thornhill *et al.* (1987) both found, as we have, that those with earlier stage tumours had sought medical advice sooner than those with late stage tumours. Possible explanations for the lack of relationship between prognostic grouping and delay have been put forward. Dixon & Moore (1953) suggested that tumours destined to metastasise do so early and Fossa *et al.* (1981) that the degree of aggressiveness of the clinical disease far outweighed the importance of delay. Host & Stokke (1959) considered that in the most malignant tumours, symptoms would be so severe that an early consultation would be likely. It is certainly feasible that slow growing tumours would be less likely to be noticed or to cause anxiety than faster growing tumours.

Correspondence: C.E.D. Chilvers.
Received 12 April 1988; and in revised form, 31 August 1988.

**Table I** Prognostic factors by delay group

| Prognostic factor | Delay (days) 0–49 | 50–99 | 100 or more | All |
|---|---|---|---|---|
| Stage | | | | |
| I | 45 (54%) | 42 (55%) | 39 (41%) | 126 (49%) |
| II | 17 (20%) | 15 (19%) | 27 (28%) | 59 (23%) |
| III and IV | 22 (26%) | 20 (26%) | 30 (31%) | 72 (28%) |
| Total | 84 (100%) | 77 (100%) | 96 (100%) | 257 (100%) |
| Markers[b] | | | | |
| Low[a] | 25 (60%) | 22 (61%) | 32 (55%) | 79 (58%) |
| High[a] | 17 (40%) | 14 (39%) | 26 (45%) | 57 (42%) |
| Total | 42 (100%) | 36 (100%) | 58 (100%) | 136 (100%) |
| Tumour volume[a] | | | | |
| Stage 1 marker-negative | 42 (50%) | 41 (53%) | 38 (40%) | 121 (47%) |
| Small | 29 (35%) | 23 (30%) | 26 (27%) | 78 (30%) |
| Large | 7 (8%) | 8 (10%) | 16 (17%) | 31 (12%) |
| Very large | 6 (7%) | 5 (6%) | 16 (17%) | 27 (11%) |
| Total | 84 (100%) | 77 (100%) | 96 (100%) | 257 (100%) |
| Prognostic group[a] | | | | |
| Stage I marker-negative | 42 (50%) | 41 (53%) | 38 (40%) | 121 (47%) |
| MRC 1–2 | 21 (25%) | 20 (26%) | 28 (29%) | 69 (27%) |
| MRC 3–5 | 19 (23%) | 13 (17%) | 18 (19%) | 50 (19%) |
| MRC 6 | 2 (2%) | 3 (4%) | 12 (13%) | 17 (7%) |
| Total | 84 (100%) | 77 (100%) | 96 (100%) | 257 (100%) |

[a]See text for definitions. [b]Excluding stage I marker-negative.

**Figure 1** Relapse-free survival from orchidectomy by delay group, excluding stage I marker-negative patients. —— delay 0–49 days, 42 cases ($O=13$, $E=7.6$); ---- delay 50–99 days, 36 cases ($O=5$, $E=6.8$); –·–·– delay > 99 days, 58 cases ($O=8$, $E=11.6$). Log rank test for trend: $\chi^2=4.39$, d.f. = 1, $P<0.05$.

**Figure 2** Relapse-free survival from orchidectomy by delay group. Stage I marker-negative patients. —— delay 0–49 days, 42 cases ($O=12$, $E=11.0$); ---- delay 50–99 days, 41 cases ($O=12$, $E=10.5$); –·–·– delay > 99 days, 38 cases ($O=8$, $E=10.5$). Log rank test for trend: $\chi^2=0.55$, d.f. = 1, $P>0.1$.

Among the patients with advanced metastatic disease studied by Scher *et al.* (1983), those who had delayed for more than 3 months were less likely to respond to treatment and were more likely to have palpable retroperitoneal disease. Oliver (1985) and Thornhill *et al.* (1987) both found that metastatic disease was more likely in those patients who had delayed longer and Thornhill *et al.* (1987) also found a significant inverse relationship between length of delay and complete remission. More advanced disease was related to delay in the MRC Working Party study (1985).

The findings with regard to survival are also inconsistent. Fossa *et al.* (1981) found no relationship between survival and delay but both Oliver (1985) and Thornhill *et al.* (1987) found an adverse effect of delay on survival. The MRC Working Party (1985) found no influence of delay on survival once stage and marker status were taken into account. In agreement with our findings, Host & Stokke (1959) found their 'survival rate' to be worse in those with the shortest period of delay.

Since we report an inverse relationship between delay and relapse-free survival it seemed appropriate to investigate whether delay might itelf be regarded as a prognostic factor. Cox regression analysis (excluding stage I marker-negative patients) indicated that, after allowing for prognostic group (MRC, 1985), delay is a significant ($P<0.05$) prognostic indicator of relapse. An alternative analysis of the prognostic effect of delay after allowing for stage and marker levels reached similar conclusions.

Although delay in seeking medical advice is in our data inversely related to relapse the explanation that we propose is that faster growing tumours are more likely to produce symptoms leading to medical consultation. The recommendation that early advice should be sought for any observed testicular changes is not invalidated by this observation. In the individual case the earliest possible medical intervention is still the best hope of catching early stage curable disease. Results of management of stage I testicular tumours are excellent with a cure rate of more than 95% (Freedman *et al.*, 1987). Raising of public awareness of this tumour by education programmes for both the general public and the general practitioner must still be the objective of health educators (Jones, 1987).

We thank Sybil Farrell for manuscript preparation. This work was supported in part by grants to the Institute of Cancer Research: The Royal Cancer Hospital from the Cancer Research Campaign and Medical Research Council, and by grants to the Royal Marsden Hospital Testicular Tumour Unit from the Bob Champion Cancer Trust.

### References

BOSL, G.J., VOGELZANG, N.J., GOLDMAN, A. & 4 others (1981). Impact of delay in diagnosis on clinical stage of testicular cancer. *Lancet*, **ii**, 970.

CUMMINGS, K.M., LAMPONE, D., METTLIN, C. & PONTES, J.E. (1983). What young men know about testicular cancer. *Prev. Med.*, **12**, 326.

DIXON, F.J. & MOORE, R.A. (1953). Testicular tumours. A clinicopathological study. *Cancer*, **6**, 427.

FOSSA, S.D., KLEPP, O., ELGJO, R.F. & 4 others (1981). The effect of patient's delay and doctor's delay in patients with malignant germ cell tumours. *Int. J. Androl.*, Supp. **4**, 134.

FREEDMAN, L.S., PARKINSON, M.C., JONES, W.G. & 5 others (1987). Histopathology in the prediction of relapse in patients with stage I testicular teratoma trated by orchidectomy alone. *Lancet*, **ii**, 294.

HOST, H. & STOKKE, T. (1959). The treatment of malignant testicular tumours at the Norwegian Radium Hospital. *Cancer*, **12**, 323.

JONES, W.G. (1987). Testicular cancer. *Br. Med. J.*, **295**, 1488.

JONES, W.G. & APPLEYARD, I. (1985). Delay in diagnosing testicular tumours. *Br. Med. J.*, **290**, 1550.

MRC WORKING PARTY ON TESTICULAR TUMOURS (1985). Prognostic factors in advanced non-seminomatous germ-cell testicular tumours: Results of a multicentre study. *Lancet*, **i**, 8.

OLIVER, R.T.D. (1985). Factors contributing to delay in diagnosis of testicular tumours. *Br. Med. J.*, **290**, 356.

PECKHAM, M.J., McELWAIN, T.J., BARRETT, A. & HENDRY, W.F. (1979). Combined management of malignant teratoma of the testis. *Lancet*, **ii**, 267.

PIKE, M.C., CHILVERS, C.E.D. & BOBROW, L.G. (1987). Classification of testicular cancer in incidence and mortality statistics. *Br. J. Cancer*, **56**, 83.

SCHER, H., CIRRINCIONE, C., BOSL, G. & 3 others (1983). Impact of symptomatic interval on prognosis of patients with stage III testicular cancer. *Urology*, **21**, 559.

THORNHILL, J.A., CONROY, R.M., KELLY, D.G. & 3 others (1986). Public awareness of testicular cancer and the value of self examination. *Br. Med. J.*, **293**, 480.

THORNHILL, J.A., FENNELLEY, J.J., KELLY, D.G., WALSH, A. & FITZPATRICK, J.M. (1987). Patients' delay in the presentation of testis cancer in Ireland. *Br. J. Urol.*, **59**, 447.